# Happenstance and regulatory culture: the evolution of innovative community mental health services in Oxfordshire in the late twentieth century

**DOI:** 10.1177/0957154X221136702

**Published:** 2022-12-19

**Authors:** Neil Armstrong, Peter Agulnik

**Affiliations:** University of Oxford, UK; Retired psychiatrist and psychotherapist, Oxford, UK

**Keywords:** Bureaucracy, historiography, institutional change, planning, regulatory culture

## Abstract

This paper uses co-produced historical material to explore the evolution of two innovative mental healthcare institutions that emerged in Oxfordshire in the 1960s. We highlight how the trajectories of both institutions were driven by chance events occurring within social environments, rather than emerging out of evidence or policy initiatives. Both institutions found a role for spontaneity and an openness to chance in the way they worked. We argue that this kind of institutional history would be unlikely today; the paper develops and uses the concept of regulatory culture to explain why. We suggest that the role of regulatory culture has been neglected in the history of psychiatry.

## Introduction

This article is one of a series of five which describe and analyse a sequence of innovative developments in mental health practice which arose in Oxfordshire in the latter part of the twentieth century. They can be shown to have drawn inspiration from the institutional reforms and therapeutic practice initiated at Littlemore Hospital Oxford following the appointment in 1959 of Dr Bertram Mandelbrote as Physician Superintendent. All can be seen as examples of unplanned ‘bottom up development’, rather than as a consequence of the implementation of national or even local policies.

The history of psychiatry can be told as a history of ideas. Conceptions of distress, coupled with technologies of care, shift and change, sometimes as a smooth, iterative sequence, sometimes in a more discontinuous manner, as a scientific revolution. Anne Harrington’s recent monograph, *Mind Fixers* (2019) is an example of this. The story she tells is of biological psychiatry superseding psychoanalytic psychiatry in the USA. The sources of change, for Harrington, are not restricted to emerging research. She discusses: commercial interests (not least a culpable entanglement with the pharmaceutical industry); professional infighting; and ‘decades of hard-nosed critique’ of such things as treatment safety and effectiveness, the logical status of disease categories and overmedicalisation (p. 272). Harrington’s work is compelling. But it perhaps downplays an additional factor, which is the role of institutions. Clinicians do not practice in isolation: the institutions in which they work, and the regulatory culture in which the institutions are placed, each play an active role in the history of psychiatry. The consequences are far reaching. Ethnographic studies have vividly demonstrated how bureaucratic expediency blurs into clinical knowledge and practice and illness experience (Brodwin, 2013; Rhodes, 1995). In the memorable phrase of Rebecca Lester (2019: 22), institutional structures become ‘folded into the ontology of illness’. For this and other reasons, the history of psychiatry is necessarily also the history of psychiatric institutions.

We describe two innovative mental health services that emerged in Oxfordshire in the 1960s: ‘The Isis Centre’, an open-door counselling and psychotherapy service, and ‘The Ley Community’, a residential recovery programme for people with severe drug and alcohol problems. We do not contextualise them as particular instances in the unfolding story of psychotherapy, or therapeutic communities. Nor do we investigate how commercial, intellectual or professional interests might have shaped them. Rather, we focus on the institutional setting. We describe a period before what Wilsdon et al. (2015) call the ‘metric tide’ washed over healthcare institutions. It feels almost prelapsarian, a time of innocence, or at least a time of less pressing moral concerns, before the ascendency of bureaucratic knowledge and its necessary simplifications, compromises and what Graeber (2015: 75) dubs ‘dead zones of the imagination’.

We suggest that the regulatory culture of the 1960s and 1970s enabled a particular variety of institution to arise, and these institutions enabled certain kinds of clinical practice. Both the Isis Centre and the Ley Community were essentially unplanned. They are bottom-up institutions, whose origins lie in chance occurrences and unexpected encounters. Each of them, at least at the start, housed clinical practice that was loose and open, flexible enough for spontaneity, to a degree close to unimaginable today (Armstrong, 2018: 186). They developed and maintained their own clinical style rather than being shaped by evidence from clinical trials or protocols designed by service managers.

These issues are raised in a recent paper reflecting on the ebb and flow of trends in mental healthcare and how we might understand the ‘quality improvement’ framework currently favoured in the NHS (Hilton, 2019). QI is based on flexible working, flattened hierarchies and an openness to innovation. As Hilton points out, this is nothing new. To a striking degree QI resembles the philosophy of therapeutic communities, just in a different guise, reframed or, perhaps, repackaged. This prompts her to ask: ‘If a successful quality improvement model existed in the UK in the middle of the 20th century, questions arise about how and why it ceased to function’ (p. 129). This is arresting not just because it unsettles an unreflective, progressive model of history. Hilton points to a wider absence of knowledge: why does one institutional form succeed another? Also, how does this relate to the style of care practised within these institutions?

This paper attempts to develop Hilton’s observations. Our historical material shows it is not just the institutions themselves that seem dated today, but the context in which they came into existence and the actions and instincts of key players. We suggest a way of theorising the flexible and open and relatively unregulated institutional environment of the 1960s and 1970s using ecological metaphors. The language of ecology suggests a dynamic relationship between setting and institution, and between institution and clinical work. This indicates a way of answering Hilton’s rhetorical question. One reason why a successful quality improvement model might be devised, improved upon and then lost lies not in the techniques themselves, or in Harrington’s vested interests, professional squabbles and critiques of psychiatry, important though they are. Instead, knowledge may be first gained and then lost because of the regulatory culture.

Not all the influence of institutional arrangements arises from formal structures and not all pertains to knowledge. Weber (2009) described being an official in a bureaucracy as a ‘vocation’ to try to capture how the bureaucratic ethos shapes individuals as moral actors. Subsequent work has sought to extend Weber’s insight. In his account of state bureaucracy in Pakistan, Hull (2012: 5) describes ‘logics, aesthetics, concepts and norms’ that go far beyond the officially demarcated frontiers of bureaucratic working. We can see these themes playing out in empirical work on healthcare. Fussinger (2011: 160), in her discussion of the history of post-war therapeutic communities, stresses the prominence of informal ‘modalities of dissemination’. In his seminal ethnography of medical school, Sinclair (1997: 16) goes further, distinguishing four institutional areas. He describes an ‘official front stage’ of lectures and ward rounds that are viewed by students and staff members. This area is clearly and intentionally institutionally shaped. Indeed, the institutional shaping is didactic: it is the formal, explicit function of medical school. But Sinclair also discusses the ‘official backstage’ where medical students work away from the gaze of staff, an ‘unofficial frontstage’ of organised activities such as sports, and the ‘unofficial backstage’ such as antics in the student bar. His point is that all four areas are institutionally shaped, drinking games as much as clinical exams. This may not always be apparent to those taking the exams or participating in the drinking games. Indeed, part of the discursive work of a bureaucracy can be to erase such effects.

So, by ‘regulatory culture’ we seek to distance our analysis from knowledge produced by the institutions we deal with. We aim to refer not just to the formal bureaucratic mechanisms that pertain to institutions, such as rules guiding the development of NHS services or constraints on funding. Rather, we refer also to the ethics, aesthetics and ontologies that surround them. These extend out from the official frontstage of clinical work into various unofficial and backstage locations. They shape an actor’s sense of how things should be done and what is an appropriate way of organising or coordinating activities. Regulatory culture influences what might be expected to be therapeutically effective, what might count as therapeutic effectiveness, and how therapeutic effects might be conceptualised. Even if it is sometimes unacknowledged and might fall under the radar of favoured research methodologies, regulatory cultures originating outside biomedicine can play a crucial role in the history of mental healthcare.

Part of our argument is that the relatively open regulatory culture of the 1960s and 1970s allowed actors to acknowledge and act upon events that were irregular, spontaneous or haphazard, more than their contemporary counterparts now might feel able to do. In terms of Lipsky’s sociology classic *Street Level Bureaucracy*, this open culture permitted officials more scope to exercise discretion (Lipsky, 2010). For Lipsky, discretion pertains to personal autonomy in decision making. In terms proposed by Onora O’Neill (2002), the actors we described we *trusted*. Our material certainly portrays actors exercising autonomy. They do not follow protocols or guidelines but respond creatively to opportunities as they emerge. The open, unplanned, egalitarian working exemplified by the development of the Isis Centre and the Ley Community was permissible at the time because it fitted the ethical, aesthetic and ontological features of the regulatory culture. More than this, the intuitive, entrepreneurial institution building that we describe below represents a way of conducting oneself that looks questionable, perhaps even unprofessional from a contemporary perspective. Our historical material suggests a close fit between regulatory culture, mental healthcare institution and treatment approaches. This might imply that the current regulatory culture, because it foregrounds the goods of accountable bureaucracy, such as transparency, efficiency, and effectiveness, favours certain kinds of institution and so forecloses certain clinical practices. But that is an argument we return to later.

Other papers presented in this issue make use of published materials and individual oral histories and witness seminars. In contrast, this paper is an exercise in co-production. Co-production in the history of psychiatry usually means the production of knowledge being shared between academics and service users, in particular to overcome stigma or to allow stakeholders to recover their own heritage (Ellis, 2017). In this case we attempt something different: the inclusion of a clinician who has a key role in the events described. Thus, Neil Armstrong is an anthropologist with an interest in bureaucracy and mental health care. Peter Agulnik is a retired NHS consultant psychiatrist and psychotherapist, who worked in Oxford for more than 40 years and who both witnessed and was a principal participant in the events we discuss. We hope that certain features of this way of working emerge as strengths in making our argument. We have already reflected on our working methods elsewhere (Armstrong and Agulnik, 2020), describing how we work together in an equitable, reciprocal way, such that the argument and the empirical material are co-created. Our intention is to work in a relatively open, unplanned way, to enable a degree of flexibility and spontaneity. We want to be cautious about post-facto rationales and justifications and attentive to the ad hoc and the improvised. Our historical writing takes two forms. When writing about the Isis Centre, Agulnik’s memory forms part of the record we draw on to produce a conventional historical narrative. In our account of the Ley Community, however, we shift to a first-person narrative. We present a quite extended piece of autoethnography in which Agulnik’s personal experiences and reflections form part of our data. We believe this method helps us acknowledge the messy, ambiguous, relational phenomenology of lived experience. One of our claims is that regulatory cultures can make it seem right to tidy away, perhaps to ignore, some of the less orderly aspects of life that we may recognise but which fall outside the authorised, official version of events. Our aim is to be less epistemically fastidious. We suggest that our improvised, interdisciplinary co-produced historiography seems well equipped to investigate life that is unplanned and unrecorded.

## The ecology of growing institutions: Oxford in the 1960s

The language of ecology suggests particular ways of thinking about the relationship between institutions and their environment. In *Mad Travellers: Reflections on the Reality of Transient Mental Illness*, Ian Hacking (1998) unpicks the relationship between certain mental disorders and their social and cultural context by thinking in terms of natural selection. He suggests that, just as in nature there are causal mechanisms linking flora and fauna and their environment, mental disorders depend on the society in which they occur and, in turn, influence that society (see also Hacking, 2007). From this position, we cannot really separate a phenomenon and its environment. In particular, the way mental disorders are classified and understood feeds back into the phenomenology of the disorder itself. In Hacking’s view some kinds of disorder (such as dissociative fugue states, of which there was an epidemic in mid-nineteenth-century Western Europe) can only exist when the conditions are right. They need what he calls an ‘ecological niche’ (Hacking, 1998: 1). If the niche disappears, the disorder goes too, as was the case with the fugue states, which more or less disappeared by the turn of the century and are seldom encountered today.

Applying Hacking’s work to mental healthcare institutions is helpful for three reasons. First, it shifts analytic focus towards the setting. If we want to understand the institutions and the care they provide, we might need to think about the context in which they evolved. In this paper we are calling part of that context the regulatory culture. Our historical material investigates this regulatory culture and thus sheds light on the institutional history of mental healthcare services in Oxford in the1960s and 1970s. Second, ecological metaphors prompt the thought that certain kinds of institution and certain kinds of clinical practice might also require a suitable ecological niche. If the environmental conditions change, for example if the regulatory culture became more restrictive in particular ways, then institutions and practices may disappear. Also, as we argued above, regulatory culture includes ethics and aesthetics, and changes in regulatory culture can make things look different. Things that seem likely or advisable or exemplary in a clinic is subject to regulatory culture. Third, an interesting feature of ecological language is that it suggests a distinctive, potentially positive role for randomness and chance. It is through random mutations that species evolve to fit their environment. In the material below, much is left to chance. Random processes, that might otherwise appear arbitrary and reflect personal agency, emerge as mechanisms through which institutions might be shaped to meet local need. A regulatory culture that permits happenstantial events thus enables a means by which institutions may evolve to fit their environment. A more restrictive regulatory culture, either by formal bureaucratic arrangements or by something looser pertaining to norms and intuitions, limits this process and thus limits a means by which institutions can adapt to local need and may lead to their downfall.

When the NHS was set up on 5 July 1948, it was conceived of as a centralised, nationalised institution that was designed to harness a rather Weberian vision of the power of rational bureaucracy. Indeed, Klein (2013: 20) remarks: ‘the NHS represented the victory of the values of rationality, efficiency and equity’. Despite this, the early decades of the NHS feel remarkably organic and unbureaucratised. Basic facts and figures were produced expressing capacity and resources, such as the number of nurses and number of beds, but data were not gathered regarding efficiency. Outcomes were not defined by bureaucrats and measured by metrics. There were no targets. Instead, it was assumed that medical professionals could be trusted to treat their patients, if they were given adequate means to do so. In this undemanding regulatory culture, bureaucratic instruments interfered very little with natural selection.

These features can be seen below. The haphazard sequences of events leading the creation of local institutions was not based on large-scale evidence demonstrating effectiveness or value for money. There was no need to conform plans to current trends in funding. Rather, need was identified locally, and chance events enabled a close fit between local need and proposed provision. The same effects are seen at a more personal level. Recognising the formative role of the environment does not displace personal intentions and efforts. Rather, chance becomes entwined with agency. Networks and happenstantial events are recognised and used by actors and produced by activity. The early inspiration or opportunity was happenstantial. Small, flexible, locally derived and locally accountable committees were formed, using personal networks, but still reliant on guesswork, ad hoc adjustments and improvisation. Part of the popularity and durability of the institutions lay in the freedom of key players to draw on the generative resources that were available in Oxford.

## The Isis Centre

The generative resources of 1960s and 1970s Oxford benefitted from a rich history that was sedimented in the present in ideas and networks and resources. This deep ecology appears to have been a particularly rich environment in which ideas might grow into institutions. Surprisingly, some of these can be traced back to Leonard and Dorothy Elmhirst and their creation of a utopian community based at Dartington Hall in Devon. The community was open and egalitarian, founded on a rejection of codified rules and making a firm commitment to the redemptive power of the Arts, and to the ideals of progressive education, increasingly prominent from the 1930s and beyond. It led to the creation of a school and college and, following a different pathway, to the creation of the glassworks that now famously bears its name.

This in itself came about through a sequence of happenstantial events. Leonard Elmhirst, the second son of a landowning North Yorkshire clergyman, was posted to India during World War I. There he met Sam Higginbotham, the founder of what is now the Indian University of Agriculture. Higginbotham inspired Leonard to become an agricultural economist and to go to Cornell University (Young, 1982/1996: 32). At Cornell, Leonard happened to meet the widowed heiress Dorothy Straight, and a romance developed. After graduation, and on her advice, Leonard returned to India to work with Rabindranath Tagore. Poet, polymath and Nobel Laureate, Tagore had sought funds from Dorothy for Santiniketan, the school and later university he had started in Bengal. When they married and set up their own educational and community organisations at Dartington, Leonard and Dorothy sought to use Tagore’s underlying philosophy of developing latent talent through learning from experience. This a continuing theme that crops up throughout this paper, recognisable in therapeutic community practice, as well as in psychoanalytic and related psychodynamic therapies.

Michael Young is a key figure in this deep ecology. As one of the earliest students at Dartington School, he became something like the adopted son of the Elmhirsts, and the continuation of their social values can be recognised in the many social institutions that he founded. His many achievements include founding The Institute of Community Studies (now The Young Foundation), The National Extension College, the forerunner of the Open University, and The Consumers Association (Briggs, 2001). He wrote prolifically, from the 1945 Labour Party Manifesto to the seminal sociological monograph *Family and Kinship in East London* (Young and Willmott, 1957) and the satire *The Rise of the Meritocracy* (Young, 1958). As fellow students at Dartington, Michael Young knew Ernest Gruenberg. After Dartington, Gruenberg qualified in medicine, and went on to train as a psychiatrist, with a particular interest in social psychiatry. He became Professor of Public Health at Johns Hopkins University. In 1957 he took an active role in tours by Medical Superintendents of New England mental hospitals interested in studying a number of pioneering British mental hospitals, following ‘Open Door’ policies (Milbank Memorial Fund, 1960). In the course of this he got to know the work of Bertram Mandelbrote, then a young medical superintendent of the Horton Road and Coney Hill mental hospitals in Gloucester.

Towards the end of the 1960s, the University of Oxford was exploring ways of housing its institutes related to agriculture under one roof. Leonard Elmhirst knew Alan Bullock, then Master of St Catherine’s College, and later Vice-Chancellor, and arranged that the Dartington Hall Trust would help to finance a joint venture with the university. This led to the building of Dartington House, which stands in a small shopping street in the central part of Oxford, next door to the main administrative building of the university. When the new building was being planned, Elmhirst approached Bertram Mandelbrote (whom he had met at a conference), to ask whether his team could make use of a suite of rooms at ground-floor level in the new building, for some form of outreach from the hospital to the community.

It was not clear at first how to use the new space. In 1970 there were no formal NHS psychotherapy services in Oxfordshire. The Warneford Hospital, with its newly created academic department under Michael Gelder, was beginning to develop a research programme using the emerging principles of behaviour therapy, and later of cognitive behaviour therapy. A Psychotherapy Department, using psychodynamic principles, opened in 1973 with the appointment of Anthony Storr. There was a sense that the rooms in Dartington House might be used in a way that responded to local emerging needs (Agulnik, Holroyd and Mandelbrote, 1976: 357; Oldfield, 1983: 7–10). A discounted rent was agreed, and a series of meetings was held with a variety of professionals, including GPs, social workers and other health and community workers. This gave a steer, and it was thought that the new Isis Centre would largely be used by the public, for obtaining information or consultation on mental health matters. The new premises also facilitated the transfer to the centre of some of the group therapy activities of the Ashurst Clinic day service at Littlemore Hospital. A coordinator, Pauline Holroyd, was appointed to respond to whatever needs were being articulated by members of the public who crossed the threshold.

The majority of people wanted to talk about difficulties in their life situations, so counselling seemed the most appropriate response. Much of this was initially provided by Pauline herself. It was not part of her job description, and at the time of her appointment there was little in the way of counselling training. But her personality and life experience, plus a background in occupational therapy, made her well suited to such activity. With increasing use came an increased need for more staff. Initially this was provided by staff from the Ashurst Clinic, which was part of the Mandelbrote-led ‘A division’. This was supplemented by others working in a range of hospital settings who had some background experience of counselling and psychotherapy, and were able to combine core roles at the hospital with work at the Centre. Voluntary Associate Counsellors augmented the service, donating their time freely in exchange for opportunities for learning. In this way a team gradually built up. Staff members met regularly for case discussion, education and supervision. Personal experience of psychotherapy became mandatory for many members of staff at the Isis Centre. In addition, some received formal training, often with organisations affiliated to the nascent British Association for Counselling and Psychotherapy. In the ensuing years, links were made with formal training courses in counselling developed jointly with Oxford Brookes University initially, and later the University of Oxford. The Centre provided many opportunities to course members for supervised practice.

A key element of the Isis Centre service was its bureaucratic autonomy. The Centre did not accept referrals, although General Practitioners sometimes recommended their patients to visit. Those who chose to make use of its services were termed clients rather than patients. Unless it was otherwise agreed, consultation was entirely private and confidential. No written reports were sent to GPs or any other health professional, and only general administrative data were gathered.

In later years, the Isis Centre spawned offshoots such as a contracted counselling service for staff members of Thames Valley Police and providing counselling services in primary care settings. The police service was provided by former Associates, with additional income used for further supervision and training. Thus, the Isis Centre came to be valued as a generator of expertise. The managerial oversight of these developments was contained entirely within the core clinical team.

All of this was contingent, flexible and open. The receipt of funding from NHS sources did not determine that the Isis Centre should adopt a particular treatment philosophy or methodology. It was not a copy of another successful institution or an instance of a scalable, evidenced intervention; there was no manual. Instead, an organic, informal piecemeal process allowed the work of Isis gradually to gain definition in response to encounters with members of the public. Even as counselling and psychotherapy began to emerge as the approaches that were the best fit for local needs, expertise was only gradually professionalised as links were established with local hospitals and universities. In the regulatory culture of the 1960s and 1970s, clinicians were trusted to make the right judgements. Their style of working was not standardised or replicable or easy to record. Documentation took a back seat to care. Informal working had certain affordances, and individual clinicians were able to respond to productive relationships and personal moments of inspiration.

## The Ley Community

One of the struggles faced by scholars working on bureaucratic cultures is how to distinguish bureaucratic self-representation from analysis (Mathur, 2016: 3). Making this distinction is problematic because institutions are constituted by their knowledge practices. They have their own episteme. It is the institution that determines what is real and what is unreal, what is visible and invisible (Mol, 2002). It is not easy to decouple oneself from this, such that one can see the invisible or engage with the seemingly unreal. David Mosse’s work on development agencies in India is a reminder of the importance of such a task (Mosse, 2005). He argues that the knowledge produced by the development organisations he analysed effectively conceals their operations (p. 230). We are not here suggesting that the Isis Centre or the Ley Community consistently misrepresent themselves, or that their primary role is to produce an authorising or self-promoting disguise. However, we do seek to describe and think through the role of regulatory culture in ways that capture the cluttered, improvised, ad hoc nature of day-to-day life, which falls outside the tidy, ordered bureaucratic episteme. To do this, we have produced a first-person, oral history narrative based on Agulnik’s personal experience.

The timing of my arrival in Oxford in 1969 was fortuitous. In the nineteen sixties there was a growing awareness that the United Kingdom had a considerable substance abuse problem. This led to a policy of rolling out residential treatment units across the country. In Oxford Regional Health Authority area, a treatment unit was to be located at Littlemore Hospital, where it became known as The Ley Clinic, named after Dr. Ley, the first medical superintendent of Littlemore. How the service was to be staffed and applied was left to local arrangements. Drug dependency, with an allocation of eleven beds, became the responsibility of Dr Mandelbrote. I, then as his senior registrar, was responsible for the psychiatric aspects of the in-patient treatment programme.

Mandelbrote was not convinced of the need for methadone prescribing which he felt could be undertaken by general practitioners. He wanted to concentrate his efforts and resources on those who were motivated, or could become motivated, to overcome their addiction. At first the programme simply continued the style of care based on democratic therapeutic community principles established within the Phoenix Unit at Littlemore in Oxford. But at the time, two new therapeutic communities had opened, Phoenix House in London and Alpha House in Portsmouth. Following the approach pioneered at Synanon in California, and subsequently modified in New York at the Phoenix House and Day Top Village programmes, these communities used a more rigorous, hierarchical approach. This became known as the ‘concept house’ model. Concept-based communities are seen as more demanding because they emphasise personal responsibility for self and others, rather than exploring underlying psychodynamic factors such as the historical experiences that led to addiction. The Oxford team established communication with both the London and Portsmouth communities.

Some rather circumstantial events gave me first-hand experience of concept-based work in the USA. After family considerations led me to leave a period of training and practice in Boston and return to England it seemed wise to keep my immigration visa open. This meant a return visit to the USA was required. It provided an opportunity to visit the Boston State Hospital Drug Dependency Unit, Day Top Village, and, crucially, I accepted an invitation to attend a weekend staff training course at Phoenix House, New York. These experiences, especially the last, made a profound impact. The existing programme at Littlemore seemed to pale into insignificance. Rather than a small community with a maximum of eleven residents, here were hundreds, living in a former penal institution on Hart Island, on Long Island sound. My personal encounter with ex-addict staff taking part in the training programme was both ego-shattering and exhilarating. These three intense days proved life-changing. It also made me realise that what we were trying to do in Oxford was naïve in the extreme. I instantly became convinced that we should adopt the same model in Oxford.

By chance the Senior Administrator of Phoenix House, London, was also attending the course. In conversation, I asked if he knew of any ex-addict who had been through that Phoenix House programme and might be interested in working in Oxford. Just a few months later he phoned me to say that an embarrassing situation had arisen. John McCabe, an ex-addict Phoenix House staff member from New York, with an otherwise good record, had been required to resign as Assistant Director following inappropriate behaviour with a female resident. Rather than sending him back to New York, he wondered whether John was the sort of person who could help the Oxford programme.

John came up to Oxford to meet Mandelbrote and the team. He was open about what had taken place in London and at a personal level we liked him. We agreed to explore whether a post could be created for him with the help of two charitable trusts. This was achieved, and he joined the team as a nursing assistant. Over the course of the next few months, and not without some pain to existing staff members, a transition was made from the Democratic to the Concept-based type of Therapeutic Community.

The hospital setting of the Ley Clinic was totally unsuitable for this type of programme. The initial plan was to find a place for a day programme, while continuing to use the hospital as a base. Happenstance again came to the rescue! On a whim I asked the Secretary at the Isis Centre whether she knew anybody who would be prepared to put up £10,000 to purchase a school house which was shortly coming up for auction in the next village. To my surprise, she replied that she had worked as an au pair for a local builder, whose firm had a charitable trust. Mandelbrote and I decided to explore this possible avenue, with the happy result of an interest-free loan. In the event, the property sold for far more than expected and was beyond our reach. This turned out to be a godsend, as within a few months, a more substantial property, which had fallen into disrepair, came onto the market. This offered the prospect of a residential programme for up to 14 people. At auction, the property, Hidsfield House, sited in an affluent residential area of Oxford, was purchased for £25,000.

An independent charitable company, The Ley Community (Oxford) Ltd., was created. The composition of the committee was important for establishing credibility with the courts and community services. Bertram Mandelbrote’s networking skills brought together the Deputy, and later Director, of the local Social Services, the Chief Probation Officer, the Chairman of the Magistrates’ Committee, and other senior magistrates, the hospital secretary, and the Treasurer of Oxford Group Homes. I was the only person with virtually no experience of committees, but was immediately elected chair, a post I held for the next 20 years. Mandelbrote provided consultant cover for both the Ley Clinic and the Ley Community. This made it possible to retain Health Service funding, whilst the charity was also able to draw on fresh sources of funding for residential support. The new structure enabled the employment of more ex-addict staff. John McCabe was appointed the first Director of the Ley Community.

By 1979 the pressure on places at Hidsfield was considerable. Again, fortuitously, a more suitable house set in seven acres of land at Yarnton, five miles from Oxford, which had recently been turned down for land development, came onto the market. The house, Sandycroft, was owned by a local medical family, the Livingstones, direct descendants of the African explorer. They took a helpful attitude to the sale, and with the increased value of Hidsfield, only £5000 was required to secure the purchase. The original loans were paid back and the Ley Community stood as a totally independent charitable organisation for over forty years. Referrals through the criminal justice system increased, and the funding structures, local initiative and a variety of further loan arrangements and selling off a portion of the seven acres of land, made it possible to build three new houses. This enabled a total residency of well over fifty places. However, changes in the regulatory culture introduced in the second decade of this century, and a considerable shift of potential funding from the criminal justice system to health and local authority budgets have led to severe disruption forcing the management committee to seek alternatives to the programme at a reduced scale.

While the Isis Centre was entirely innovative in its conception, the Ley Community to a considerable extent relied on existing models established at Synanon in California and developed by Phoenix House in New York. With the rising tide of drug addiction being experienced in the UK in the 1960s, new services were being developed, under central government initiatives. New financial resources were being made available to regional drug and alcohol services throughout the country. But the institutional history of the Ley Community is nonetheless a history of discretion: chance events, opportunities seized, networks explored. Agulnik had inspiring experiences in New York because he needed to keep his visa open. But the regulatory culture was such that he was able to bring the ideas back to Oxford. Small, nimble, responsive institutional structures, such as the Ley Community charitable company, exercised considerable autonomy. The Ley Community evolved without needing to conform to the demands of policy trends. Indeed, the transition from democratic to concept-type community reflected an internal process.

The ethical qualities of the regulatory culture are particularly striking in this case. This reliance on trust and personal connections that comes across as dynamic and responsive might look also like patronage and privilege. A lot is left to the judgement of the key players. There were no bureaucratic instruments in place to demonstrate transparency or accountability or diversity. Similarly, care is flexible and spontaneous, leaving staff with a great deal of discretion. These are not the institutional preconditions of a standardised healthcare service delivering care that can de represented bureaucratically as safe or efficient. There is no way of avoiding post-code lotteries. The ambivalence is acute in the case of McCabe. Relocating him might have looked like institutions refusing to be accountable, insisting on defending their own. Had McCabe crossed another red line in Oxford, that interpretation would be hard to resist. But from the ethical imaginary of the time, it looked rather different, something like another instance of entrepreneurial, flexible practice, keeping things in proportion, and making the best of the available resources.

## Regulatory cultures and mental healthcare

Both these examples demonstrate the salience of regulatory culture, not just as a set of formalised rules and regulations, but as a broader assemblage of ethics and aesthetics, shaping individual conduct, notions of possibility and propriety, and through this, modalities of care and illness experience. The relaxed regulatory culture of the 1960s and 1970s allowed well connected, entrepreneurial individuals to make the most of what was probably an unusually rich generative environment in Oxford. Many of the events described above contain an element of chance: social connections, happenstantial events, personal judgements and intuitions. Local initiative was seen as the right way to use informal networks. The key players in such a setting are individuals like Mandelbrote, skilled networkers whose ingenuity and agility enable them to make the most of opportunities and resources. Institutions could then grow and change, evolving to fit local needs. The clinical working style at both institutions was thus arrived at by local deliberation rather than higher-level policy or evidence bases. This can be framed as undesirable or improper, even unethical. Also, we recognise that this organic freedom can lead to trajectories rather different from those of the Ley Community and the Isis Centre. Spandler and Carr (2022) describe how the (now discredited) aversion therapy for lesbian and bisexual women was made possible by the space for discretion opened up by relaxed regulatory cultures of 1960s mental healthcare. But thinking of good practice in terms of conformity to guidelines or defended by evidence from trials reflects a current, highly bureaucratised moral imaginary. The ethical intuitions that tie professionalism to adherence to protocols rather than, say, personal judgement, do not derive from philosophies of care, but from the regulatory culture. Ecological metaphors show a potential cost of more restrictive, intrusive regulatory cultures because they interfere with natural selection. Chance can be valuable because it makes links between local needs and preferences and local institutions, and can be stymied by intrusive regulations or a sense that such activity is improper. Regulatory cultures that are less intrusive permit a certain entrepreneurial dynamism. Happenstance becomes a kind of resource. The ideas and energies of creative individuals can lead to innovation.

Earlier in this paper we discussed Hilton’s account of QI and the way that ideas might arise and be developed, only to disappear again. The notion of regulatory culture helps us understand this seemingly irrational process. Through the 1970s and 1980s, what Marilyn Strathern (2000) calls ‘audit cultures’ began to spread, bringing with them bureaucratic technologies known as performance indicators that documented the relationship between inputs and outputs. Between 1991 and 1997, in a series of reforms, ‘internal markets’ were introduced by separating the purchase of care from the provision of care. This meant making hospitals independent of health authorities and creating competition between them and private providers. Markets require information. Further bureaucratic mechanisms were devised to produce data that could be compared so that competitive tendering became possible. In this new regulatory culture, evolutionary processes were disrupted and top-down, evidence-based planning became an ethical imperative. From such a standpoint, evolutionary institutional development seems risky in a culture where risk is censured.

The impact of changes in the regulatory culture on the Isis Centre were dramatic. In 2017 the decision was made between Oxford University and the (now responsible) Oxford Health NHS Trust not to renew the lease. The service was moved to a suite of rooms in a building which had once been part of the Cowley Road Hospital. Typical NHS service signage provides an entirely different ambience, while institutional autonomy is lost. The Ley Community has faced an even more tempestuous trajectory. Changes to funding structures pushed it towards the public health budget and away from the criminal justice system, which might have been in a better position to fund it.

The Ley Community looks like an outlier when viewed from within the regulatory culture; it does not deliver a standardised, recordable intervention, but seeks personal moral change: a transformation of values, commitments and priorities. In her account of Paddington Day Hospital, Helen Spandler (2006: 145) suggests that if therapeutic communities have a chaotic aspect, it need not mean they are in a state of disorder, because ‘TC knowledge is insecure, unstable, shifting, uncertain and, most importantly, contested’. Spandler is surely right when she argues that TC knowledge is unsuited to more searching regulatory regimes. We suggest that evolved institutions promote a professional style or persona that is suited to therapeutic communities. For example, the ‘communalism’ that Rapoport (1960: 62) regarded as an essential feature of therapeutic communities demands a high degree of openness and egalitarianism from staff members. Kennard writes of therapeutic communities as an egalitarian learning process, intrinsically open and reactive (Kennard, 1998: 27). In their distillation of the ‘quintessence’ of therapeutic communities, Pearce and Haigh (2017: 53–61) describe complex processes that require sensitivity, flexibility and spontaneity from staff members. These qualities fall against the grain of more bureaucratised working, both in terms of explicit practice and in matters of ethics and norms. They are less legible and rely on personal judgement based on immediate context.

## Discussion

This paper has tried to argue for increased attention to be given to the role of institutions and the agency of regulatory culture in the history of psychiatry. We have drawn attention to how the permissive regulatory culture of the 1960s turned unforeseen happenstantial events into a sympathetic ‘ecological niche’. Both the Isis Centre and the Ley Community arose and grew because of facilitating environmental factors. New developments in the field of mental health care were derived from bottom-up innovation. To use a botanical metaphor, the germ of an idea needs to find a responsive environment so that a potentially viable seed is formed, but which requires the right conditions if it is to germinate, grow and reproduce. We have cited here two descriptive examples. Others, such as the creation of Restore or the Elmore Community Support Team, described elsewhere in this issue, could have been used.

Thinking of institutions in this way disrupts simple progress narratives and highlights the risks of authorising institutional discourse entering academic analysis. This is because regulatory cultures include modes of justification. To use Gayatri Spivak’s phrase, our analysis falls *against the grain* of institutional knowledge because it brings into the analysis factors such as affect and aesthetics that complicate and potentially compromise institutional self-authorisation or self-promotion (Spivak, 1988: 271–313; our italics). The world of the 1960s and 1970s placed more trust in clinical judgement as a source of authority and less in fidelity to authoritative protocols. Clinicians had autonomy and could exercise discretion. From such a viewpoint, advances in research or developing ideas look secondary and reactive. The pressures on the Isis Centre and the Ley Community do not indicate evidence of absence of positive effects, or absence of evidence of effects, so much as changes in regulatory culture that reframe flexible, unbureaucratised working as an increasingly outlandish and style of working that is hard to justify. Styles of work that represent themselves as ad hoc and flexible are vulnerable, while those that appear to be precise, scalable and legible appear legitimate.

There may be reasons for clinicians to be cautious about more demanding regulatory cultures. How important to a mental healthcare clinician are the qualities of being dispassionate and impartial? Is impersonal precision the right goal when trying to empower distressed individuals? Ballatt and Campling (2011: 125) put it like this: ‘The way in which the tasks, priorities, anxieties and relationships are viewed and managed makes up the culture of an organisation and this is, in turn, *internalised* by everyone concerned’ (original emphasis). The regulatory culture is not derived from clinical expertise, but is part of a wider set of processes. It need not coincide with clinical goals. This raises a question: to what degree should the ethos of a clinician resemble the ethos of a bureaucrat? Ballatt and Campling (p. 44) suggest that the institutional culture of the NHS has, because of excessive regulation and intrusive managerialism, largely excluded kindness from healthcare. This, in their view, is not just a matter of patient approval or the acceptability of care. Rather, care without kindness is less effective. What they call ‘intelligent kindness’ leads to symptomatic improvement, wellbeing and satisfaction.

Much research in mental healthcare has focussed on interventions. Less attention has been given to the regulatory culture and the resulting professional practice and personae. This may have come at a cost. What our historical material suggests is that changes in the regulatory culture have had a decisive effect on both the institutional form of mental healthcare and the work of mental healthcare professionals. This contributes more broadly to the historiography of mental health. As well as emerging research evidence, the pursuit of commercial or professional interests, and disputes between proponents and critics of the current status quo, we need to consider regulatory culture.
